# Book Reviews

**Published:** 1873-03-01

**Authors:** 


### Book Reviews.

Fistula, Hæmorrhoids, Painful Ulcers, Stricture, Pralasus, and other Diseases of the Rectum, their Diagnoses and Treatment. By William Allingham, Fellow of the Royal College of Surgeons of England, etc., etc. Second edition, revised and enlarged. Philadelphia: Lindsay & Blakiston. 12mo., 265 pages.

The extended title of this little work sufficiently explains its contents. The author has condensed into it the results of his many years experience and observation in the treatment of diseases of the rectum.

A Handbook of Therapeutics. By Sidney Ringer, M.D. Third edition. New York: Wm. Wood & Co. 8vo., 560 pages.

A concise, practical, but comprehensive work; it fully meets the requirements of the class of students and young practitioners, for whom it is more especially designed.

Surgical Diseases of Infants and Children. By M. P. Guersant, Honorary Surgeon to the Hospital des Enfants Malades, Paris, etc. Translated from the French by Richard J. Dunglison, M.D. Philadelphia: H. C. Lea, Publisher. For sale by Jansen, McClurg, & Co., 117 and 119 State street, Chicago. 8vo., 350 pages.

In a note appended to the preface, the translator says that, "In offering to the American reader a translation of this valuable work of M. Guersant, it has been deemed expedient to preserve, without note or comment, the original text of one whose authority on the surgical affections of early life is so generally recognized." As allusion is liberally made to the experience of other French writers and practitioners on almost all the subjects discussed, this treatise on the surgical diseases of children may be justly regarded as a reflection of the views of the most distinguished surgeons of that country, in the very interesting class of cases embraced in it.

A Treatise on the Theory and Practice of Obstetrics. By Wm. H. Byford, A.M., M.D. Second Edition; thoroughly revised. New York: Wm. Wood, & Co. For sale by W. B. Keen & Cook, Chicago.

The call for a second edition of this work, so soon after its first issue, is the best possible evidence of the general favor with which it has been received. The author's endeavor to supply a plain, practical, and concise text book on this important branch of medicine, seems to have been fully appreciated by the profession.

The Practice of Surgery. By Thomas Bryant, F. R. C. S., Surgeon to Guy's Hospital, with 507 illustrations. Philadelphia: H. C. Lea, Publisher. For sale by Jansen, McClrg, & Co., Chicago.

Will be reviewed in our next number.

First Annual Report of the Supervising Surgeon of the Marine Hospital, Service of the United States, for the year 1872, containing a brief historical sketch of the service from the date of its organization in 1798. Government Printing Office, Washington.

Fœticide or Criminal Abortion. A Lecture Introductory to the Course on Obstetrics and the Diseases of Women and Children; University of Pennsylvania. By Hugh L. Hodge, M.D. Fourth Edition. Philadelphia: Lindsay & Blakiston. Small, 12mo., 55 pages. Price, 30 cents.

